# HPV16 E1 dysregulated cellular genes involved in cell proliferation and host DNA damage: A possible role in cervical carcinogenesis

**DOI:** 10.1371/journal.pone.0260841

**Published:** 2021-12-30

**Authors:** Fern Baedyananda, Arkom Chaiwongkot, Shankar Varadarajan, Parvapan Bhattarakosol

**Affiliations:** 1 Applied Medical Virology Research Unit, Department of Microbiology, Faculty of Medicine, Chulalongkorn University, Bangkok, Thailand; 2 Division of Virology, Department of Microbiology, Faculty of Medicine, Chulalongkorn University, Bangkok, Thailand; 3 Department of Molecular and Clinical Cancer Medicine, Institute of Systems, Molecular and Integrative Biology, University of Liverpool, Liverpool, United Kingdom; Istituto Nazionale Tumori IRCCS Fondazione Pascale, ITALY

## Abstract

HPV16 is the most prominent cause of cervical cancer. HPV16 E1, a helicase required for HPV replication exhibits increased expression in association with cervical cancer progression, suggesting that E1 has a similar effect on the host as the HPV16 E6 and E7 oncoproteins. This study aimed to determine whether expression of HPV16 E1 correlated with carcinogenesis by modulating cellular pathways involved in cervical cancer. HEK293T cells were transfected with pEGFP, pEGFPE1 or truncated forms of HPV16 E1. Cell proliferation, cell death, and the impact of HPV16 E1 on host gene expression was then evaluated. HPV16 E1 overexpression resulted in a significant reduction of cell viability and cellular proliferation (*p*-value<0.0001). Moreover, prolonged expression of HPV16 E1 significantly induced both apoptotic and necrotic cell death, which was partially inhibited by QVD-OPH, a broad-spectrum caspase inhibitor. Microarray, real time RT-PCR and kinetic host gene expression analyses revealed that HPV16 E1 overexpression resulted in the downregulation of genes involved in protein synthesis (RPL36A), metabolism (ALDOC), cellular proliferation (CREB5, HIF1A, JMJDIC, FOXO3, NFKB1, PIK3CA, TSC22D3), DNA damage (ATR, BRCA1 and CHEK1) and immune response (ISG20) pathways. How these genetic changes contribute to HPV16 E1-mediated cervical carcinogenesis warrants further studies.

## Introduction

Almost all cervical cancer cases and a significant proportion of other cancers are attributed to human papillomavirus (HPV) infection [1]. HPV16 is the most common type of HPV associated with cervical cancer as well as other HPV related cancers [2]. HPV is a small naked icosahedral DNA virus belong to Family *Papillomaviridae*. The circular double stranded DNA consists of 6 early genes (E1, E2, E4, E5, E6, E7) and 2 late genes (L1 codes major capsid protein and L2 codes minor capsid protein) [3]. E1, a replication helicase protein and the DNA binding protein E2, are required for viral genome replication. E1 and E2 form complexes which facilitate the binding of replication factors to the viral origin of replication [4]. In addition, E2 acts as a transcriptional regulator for viral gene expression [5]. A hallmark of cervical cancer is viral integration into host genome which often results in the disruption of the HPV E2 ORF. Following integration, the E2 gene is disrupted and there is an increased expression of the integrated viral oncogenes, which are no longer regulated by E2 [6]. The early viral genes that are retained in integrated HPV genome include E6, E7 and E1 [7]. The E6 protein of high-risk HPV binds and degrades the tumor suppressor protein p53, which is a crucial genome maintenance protein. Degradation of p53 by E6 causes cells to proliferate unchecked and apoptosis is decreased [8]. E7 binds to the retinoblastoma protein (pRb), a tumor suppressor protein that acts to bind and inhibit the transcription factor E2F. Binding of E7 to pRb causes E2F to be released and drives cell proliferation [3]. In addition to expression of E6 and E7, which decrease apoptosis and increase cell cycle activity, the E1 helicase may also promote DNA damage and drive cell proliferation [9].

Carcinogenesis is caused through aberration of many cellular pathways, including inhibition of cell death, increased cell proliferation and DNA damage [10]. Recently, studies have pointed that the E1 protein, which is responsible for the replication of HPV, could play a role in carcinogenesis [11] and DNA damage [9]. Moreover, we have previously demonstrated that elevation of E1 expression correlates with cervical cancer progression [12]. However, whether E1 plays a role in cancer development is still unknown. The present study aimed to determine whether HPV16 E1-mediated cervical cancer progression accompanies changes in host cellular pathways that could be attributed to carcinogenesis.

## Materials and methods

### Cell culture

Human embryonic kidney (HEK) 293T cell line, purchased from ATCC (USA), was grown in Dulbecco’s Modified Eagle’s Medium (DMEM, GE Healthcare Life Sciences, USA), supplemented with 10% heat-inactivated fetal bovine serum (FBS; GIBCO Thermo Fisher, USA), 100 unit/ml penicillin and 100 μg/ml streptomycin (GIBCO Thermo Fisher, USA) and 1mM of sodium pyruvate (GIBCO Thermo Fisher, USA) and incubated at 37°C, 5% CO_2_. The cells were passaged when 80–90% confluence was reached.

### Plasmids and reagents

Plasmids used contained various domains of codon optimized HPV16 E1were generated by the addition of stop codons into the HPV16 E1 sequence. These plasmids were kindly provided by Dr. Seiichiro Mori (National Institute of Infectious Diseases, Japan) [13]. The plasmids were subcloned into pEGFP-C1 plasmid (Clontech, USA) at the XhoI and KpnI restriction sites, using the forward primer: 5’- CTC CTC CTC GAG CTG CCG ACC CCG CTG GGA CG-3’ and reverse primer: 5’-GGT GGT GGT ACC CTA CAG TGT GTT GGT ATT TTG ACC-3’. The final plasmids used in this study were: pEGFP-E1-184, pEGFP-E1-359, pEGFP-E1-439 (expressing amino acids 1–184, 1–359, and 1–439 of HPV16 E1, respectively) pEGFP-E1 (full length HPV16 E1) and pEGFP (vector control). Q-VD-OPH [5-(2,6-Difluorophenoxy)-3-3-methyl-1-oxo-2-(2-quinolinylcarbonyl)aminobutylamino-4-oxo-pentanoic acid hydrate], a pan caspase inhibitor was purchased from Sigma Aldrich, USA.

### HPV16 E1 transfection and detection

HEK 293T cells were transfected with either pEGFP, pEGFP-E1, pEGFP-E1-184, pEGFP-E1-359, or pEGFP-E1-439 using X-tremeGENE HP DNA Transfection reagent (Roche, USA). Transfection was performed according to manufacturer protocol. Complexes were prepared in Opti-MEM™ I Reduced Serum Media (GIBCO Thermo Fisher, USA). A 1:3 ratio of plasmid to transfection reagent was used for all experiments at a concentration of 1 μg plasmid/100 μL Opti-MEM™. GFP positive cells were detected using fluorescent microscopy (Olympus FV 3000, Japan) and flow cytometry (BD FACSAria™ II, USA).

### Cell proliferation assays

HEK 293T cells were seeded at a density of 5x10^4^ cells/mL into each well of 24-well plate. After 24 hours, cells were transfected with 0.25 μg of either pEGFP, pEGFP-E1, pEGFP-E1-184, pEGFP-E1-359, or pEGFP-E1-439. At 12–48 hours post-transfection, cells were harvested and stained with 0.4% trypan blue (Sigma-Aldrich, USA). Viable and non-viable cells were counted using a hemocytometer counting chamber. Two independent experiments with triplicate wells were performed. Alternatively, cells transfected as above were harvested with 10μL of CountBright™ Absolute Counting Beads (Invitrogen, USA) and cell count was determined by $\frac{AxC}{B}$, in which A = number of cell events, B = number of bead events and C = assigned bead count of the lot (beads/10μL). Cell proliferation using TetraZ™ Cell Proliferation Kit (BioLegend, USA) was performed by incubating cells, transfected as above, with 10μL of TetraZ™ solution at 37°C for 2 hours. Absorbance was measured at 450nm using a microplate reader **(**Perkin Elmer, USA**).**

### Cell viability assays

To determine the cytotoxicity of E1 transfected cells, Zombie Yellow™ Fixable Viability Kit (BioLegend, USA) was used on cells transfected as above. Cells were incubated with 100 μL of diluted Zombie Yellow™ (1:100 in PBS) solution at room temperature, in the dark, for 15 minutes. After incubation, the cells were washed and resuspended in PBS containing 2% FBS and then analyzed by flow cytometry (BD FACSAria^™^ II, USA). Two independent experiments with triplicate wells were performed. To quantify the extent of apoptosis, cells were resuspended in 100 μL of binding buffer (140 mM NaCl, 4 mM KCl, 0.75 mM MgCl_2_, 10 mM HEPES and 2.5 mM CaCl_2_) and stained with 5 μL of APC conjugated annexin V (Biolegend, USA) in the dark at room temperature for 15 minutes. Propidium iodide (final concentration 2.5 μg/mL) (Biolegend, USA) was added to the cells and incubated for 5 minutes at room temperature in the dark and analyzed by flow cytometry (BD FACSAria^™^ II, USA). For confocal imaging of apoptosing cells, cells were incubated with 5 μL of APC conjugated annexin V for 15 minutes and imaged (Olympus FV 3000, Japan).

### RNA extraction for microarray analysis

GFP positive HEK 293T cells, transfected with either pEGFP, pEGFP-E1, were sorted by flow cytometry (BD FACSAria^™^ II, USA), and DNA, RNA, and protein from the sorted cells were extracted using NucleoSpin® TriPrep (Macherey-Nagel, Germany). Extracted RNA was then precipitated using 0.3M sodium acetate and 1 volume of isopropanol. The solution was chilled at -20°C for at least 1 hour. The pellet was centrifuged for 15 minutes (15,000 g, 4°C), washed with 70% ethanol and centrifuged again for 15 minutes (15,000 g, 4°C). Precipitated RNA was then sent for microarray analysis (Macrogen, Republic of Korea).

### Microarray analysis

RNA purity and integrity were evaluated by ND-1000 Spectrophotometer (NanoDrop, Wilmington, USA), Agilent 2100 Bioanalyzer (Agilent Technologies, Palo Alto, USA). Total RNA was amplified and purified using TargetAmp-Nano Labeling Kit for Illumina Expression BeadChip (EPICENTRE, Madison, USA) to yield biotinylated cRNA according to the manufacturer’s instructions. Briefly, 400 ng of total RNA was reverse-transcribed to cDNA using a T7 oligo(dT) primer. Second-strand cDNA was synthesized, in vitro transcribed, and labeled with biotin-NTP. After purification, the cRNA was quantified using the ND-1000 Spectrophotometer (NanoDrop, Wilmington, USA). 750 ng of labeled cRNA samples were hybridized to each Human HT-12 v4.0 Expression Beadchip for 17h at 58°C, according to the manufacturer’s instructions (Illumina, Inc., San Diego, USA). Detection of array signal was carried out using Amersham fluorolink streptavidin-Cy3 (GE Healthcare Bio-Sciences, Little Chalfont, UK) following the bead array manual. Arrays were scanned with an Illumina bead array Reader confocal scanner according to the manufacturer’s instructions.

### Raw data preparation and statistical analysis for microarray

The quality of hybridization and overall chip performance were monitored by visual inspection of both internal quality control checks and the raw scanned data. Raw data were extracted using the software provided by the manufacturer (Illumina GenomeStudio v2011.1 (Gene Expression Module v1.9.0)). Array probes were transformed by logarithm and normalized by quantile method. Statistical significance of the expression data was determined using fold change. For a DEG set, Hierarchical cluster analysis was performed using complete linkage and Euclidean distance as a measure of similarity. Gene-Enrichment and Functional Annotation analysis for significant probe list was performed using Gene Ontology (http://geneontology.org) and KEGG pathway (http://kegg.jp). All data analysis and visualization of differentially expressed genes was conducted using R 3.0.2

### Validation of host gene expression

GFP positive HEK 293T cells, transfected with either pEGFP or pEGFP-E1, were sorted by flow cytometry (BD FACSAria™ II, USA), and DNA, RNA, and protein from the sorted cells were extracted using NucleoSpin® TriPrep (Macherey-Nagel, Germany). Extracted RNA (3 μg) was reverse transcribed using Super Script IV (Invitrogen, USA) according to manufacturer protocol. The cDNA from pEGFP or pEGFP-E1 transfected cells was amplified by real-time PCR using PrimePCR™ 96 well as follows: 10μL of 2x SsoAdvanced™ Universal SYBR® Green Supermix (Bio-Rad, USA), 1μL of cDNA template, and 9μL of Nuclease-free H_2_O for a final volume of 20μL. The PCR reaction was as follows: initial denaturation at 95°C for 2 minutes, followed by 40 cycles of 95°C for 30 seconds, 60°C for 30 seconds. GAPDH was used as a reference gene. Gene expression analysis was calculated using the 2^-ΔΔCT^ method.

## Results

### Full-length HPV16 E1 decreases cellular proliferation

**Fig 1:**
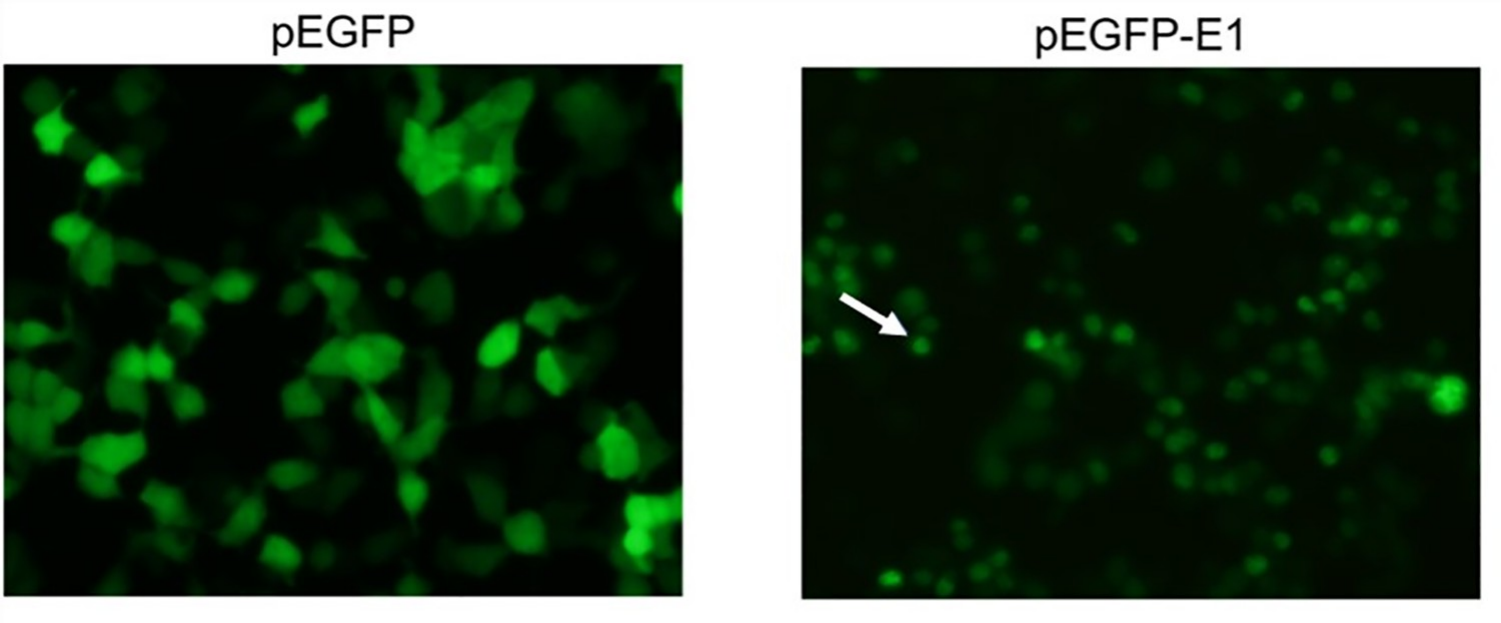
pEGFP and pEGFP-E1 transfected HEK 293T cells. Green fluorescence was observed in HEK 293T cells transfected with pEGFP alone and with pEGFP-E1 transfected cells under fluorescence microscopy. Arrow denotes nuclear localization.

**Fig 2:**
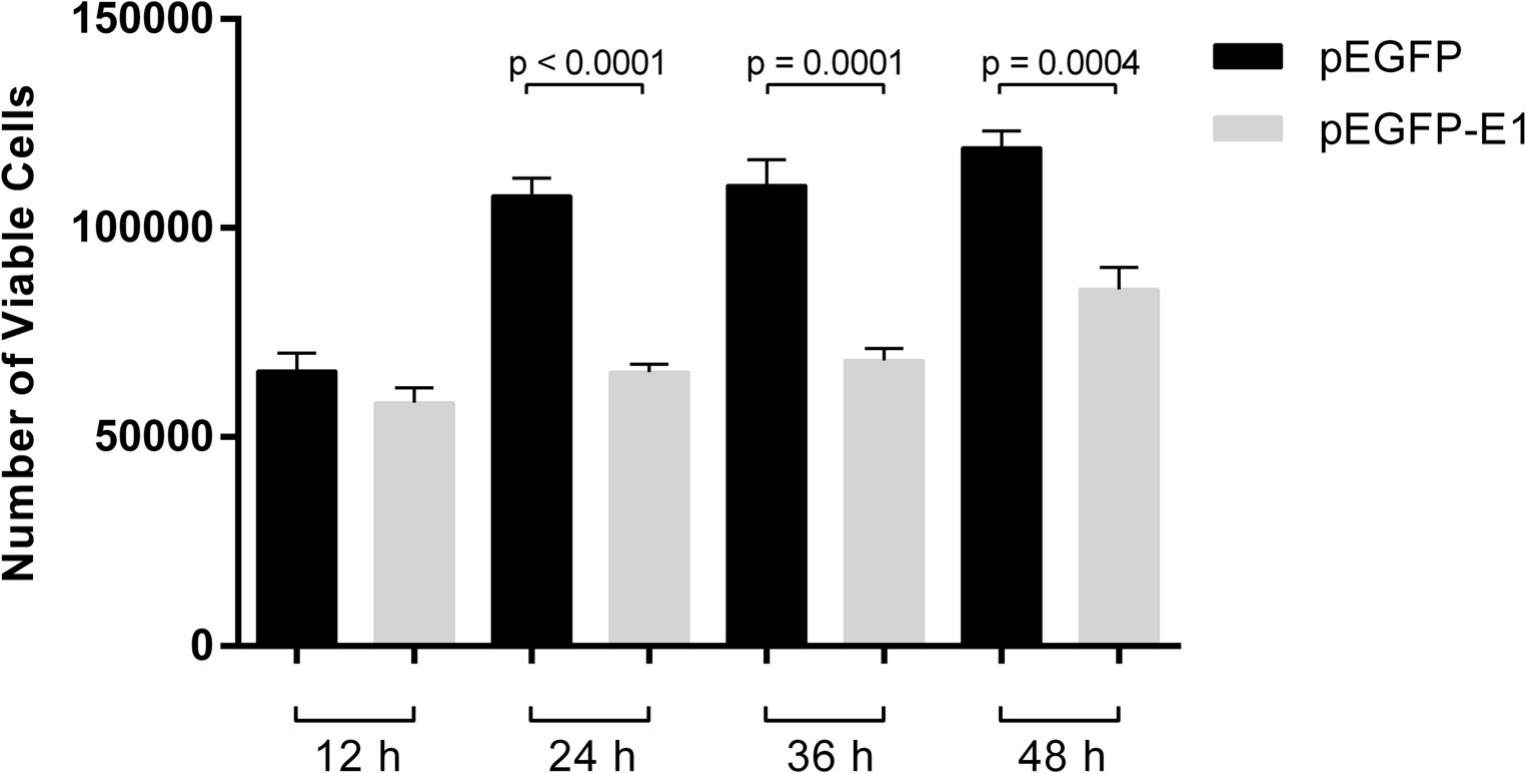
HPV16 E1 decreases cell growth. Cell viability was quantitated using trypan blue. Statistically significant cell growth reduction was observed in pEGFP-E1 transfected cells at 24, 36, and 48 hours post-transfection.

In order to determine the role of HPV16 E1, HEK 293T cells were transfected with either pEGFP (vector control) or pEGFP-E1 (EGFP tagged HPV16 E1). The green fluorescence in pEGFP-E1 transfected HEK 293T cells was found mainly localized in the nucleus, whereas the green fluorescence was observed both in the cytoplasm and nucleus of pEGFP transfected cells (Fig 1). Moreover, HPV16 E1 overexpression reduced cell growth significantly by 39%, 38%, and 28% at 24-, 36- and 48-hours post-transfection, respectively (Fig 2).

**Fig 3:**
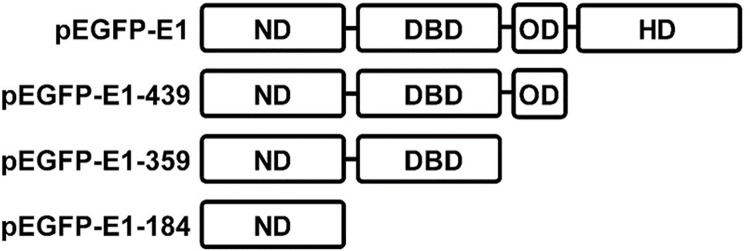
Truncated pEGFP-E1 plasmids and corresponding domains. ND: N-terminal domain, DBD: DNA binding domain, OD: oligomerization domain, HD: ATPase helicase domain.

**Fig 4:**
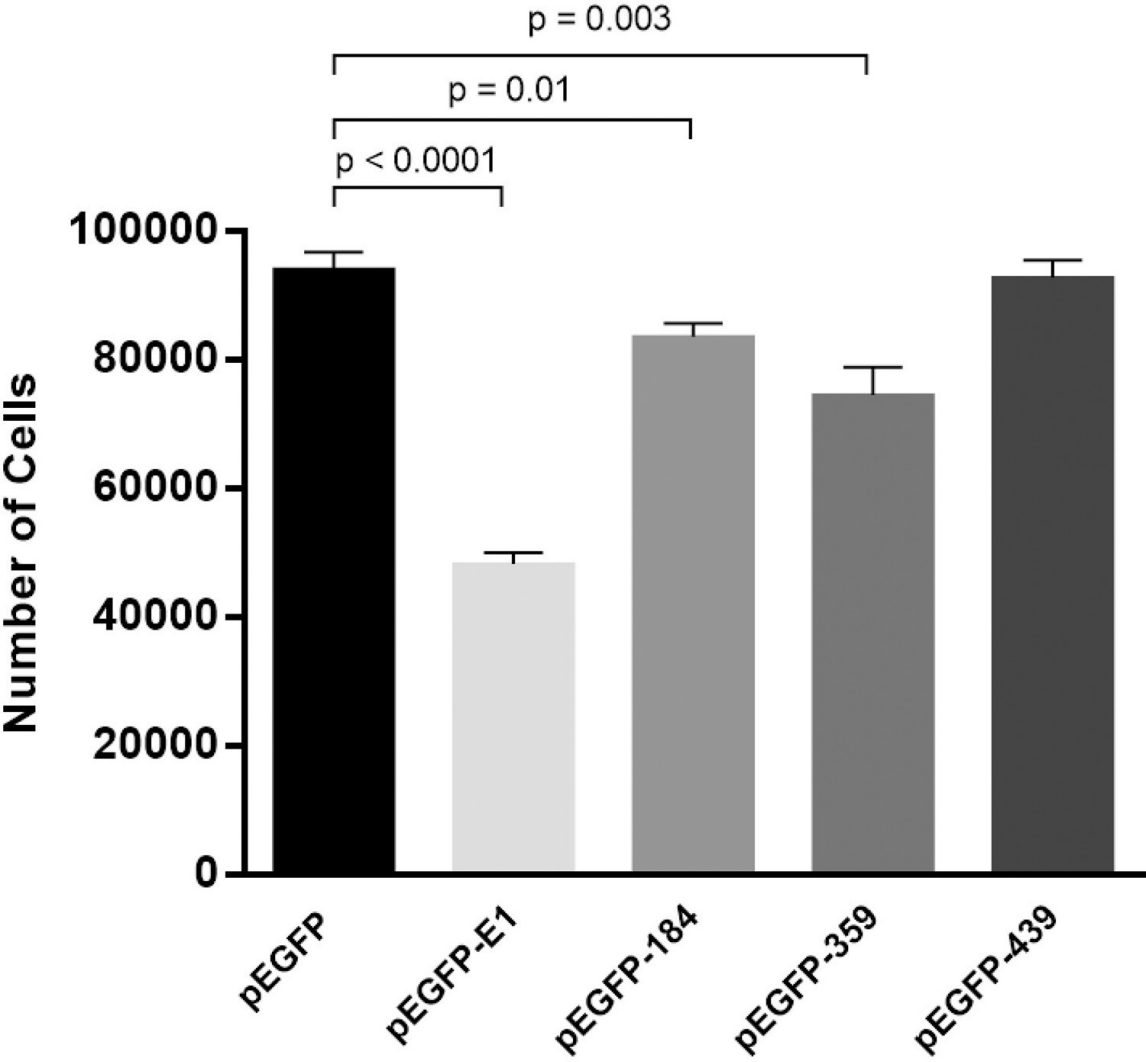
Full-length and partially truncated forms of E1inhibit cell proliferation. HEK 293T cells were transfected with either the vector control (pEGFP), full-length HPV16 E1(pEGFP-E1) or one of the truncated forms (pEGFP-E1-184, pEGFP-E1-359, and pEGFP-E1-439). Cell count was analysed using CountBright™ beads and flow cytometry.

HPV16 E1 is comprised of 4 main domains: N-terminal domain (ND), which contains the nuclear localization and export signals; the DNA binding domain (DBD), which binds to the viral replication origin; and the oligomerization domain (OD), which is responsible for oligomerization and the ATPase helicase domain (HD). To identify the domains that are responsible for cell growth reduction, plasmids containing the various domains of E1, namely pEGFP-E1-184 (ND), pEGFP-E1-359 (ND+DBD), pEGFP-E1-439 (ND+DBD+OD), pEGFP-E1 (full-length E1) (Fig 3) and pEGFP (vector control) were overexpressed in HEK 293T cells and cell growth measured using CountBright™ absolute counting beads and flow cytometry. A statistically significant reduction in cell growth at 24 post-transfection was observed in pEGFP-E1 (49%, *p*-value < 0.0001), pEGFP-E1-184 (11%, *p*-value < 0.01) and pEGFP-E1-359 (21%, *p*-value < 0.003), compared to pEGFP vector control (Fig 4).

**Fig 5:**
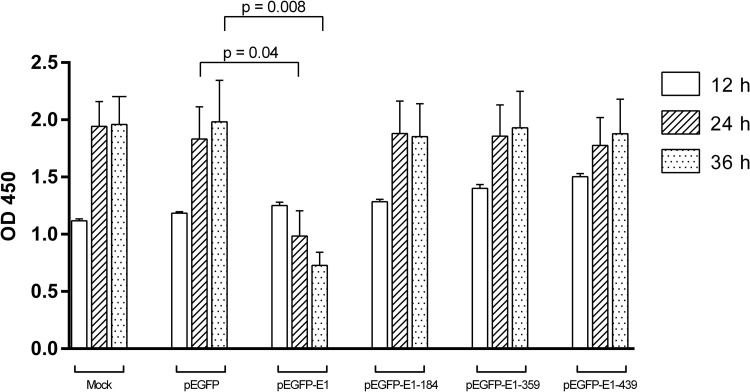
Cell proliferation was significantly decreased in full-length HPV16 E1 (pEGFP-E1) transfected cells. Cell proliferation of pEGFP, vector control, full-length HPV16 E1 (pEGFP-E1) and truncated forms of HPV16 E1 (pEGFP-E1-184, pEGFP-E1-359, and pEGFP-E1-439).

To confirm the effect of full-length and truncated forms of E1, cell proliferation was quantitated by The TetraZ™ Cell Counting Kit, a colorimetric cell counting kit, based on the ability of viable cells to convert tetrazolium salt to formazan. The results showed that there was an increase in cell proliferation with incubation time for all truncated forms of HPV16 E1 and pEGFP. In contrast, full-length HPV16 E1 showed a significant reduction in cell proliferation at 24 (*p*-value = 0.04) and 36 (*p*-value = 0.008) hours post-transfection, compared to the pEGFP vector control. The mean OD450 of pEGFP vector at 24 and 36 hours, post-transfection was 1.83 and 1.98, whereas the mean OD450 for E1 transfected cells was 0.98 and 0.72 (Fig 5). Taken together, the results concluded that full-length E1 strongly inhibited cell proliferation at a statistically significant level in all experiments. It was also noted that only cells transfected with full-length HPV16 E1 exhibited decreased cell viability (Fig 5).

### HPV16 E1 induces apoptosis and necrosis

**Fig 6:**
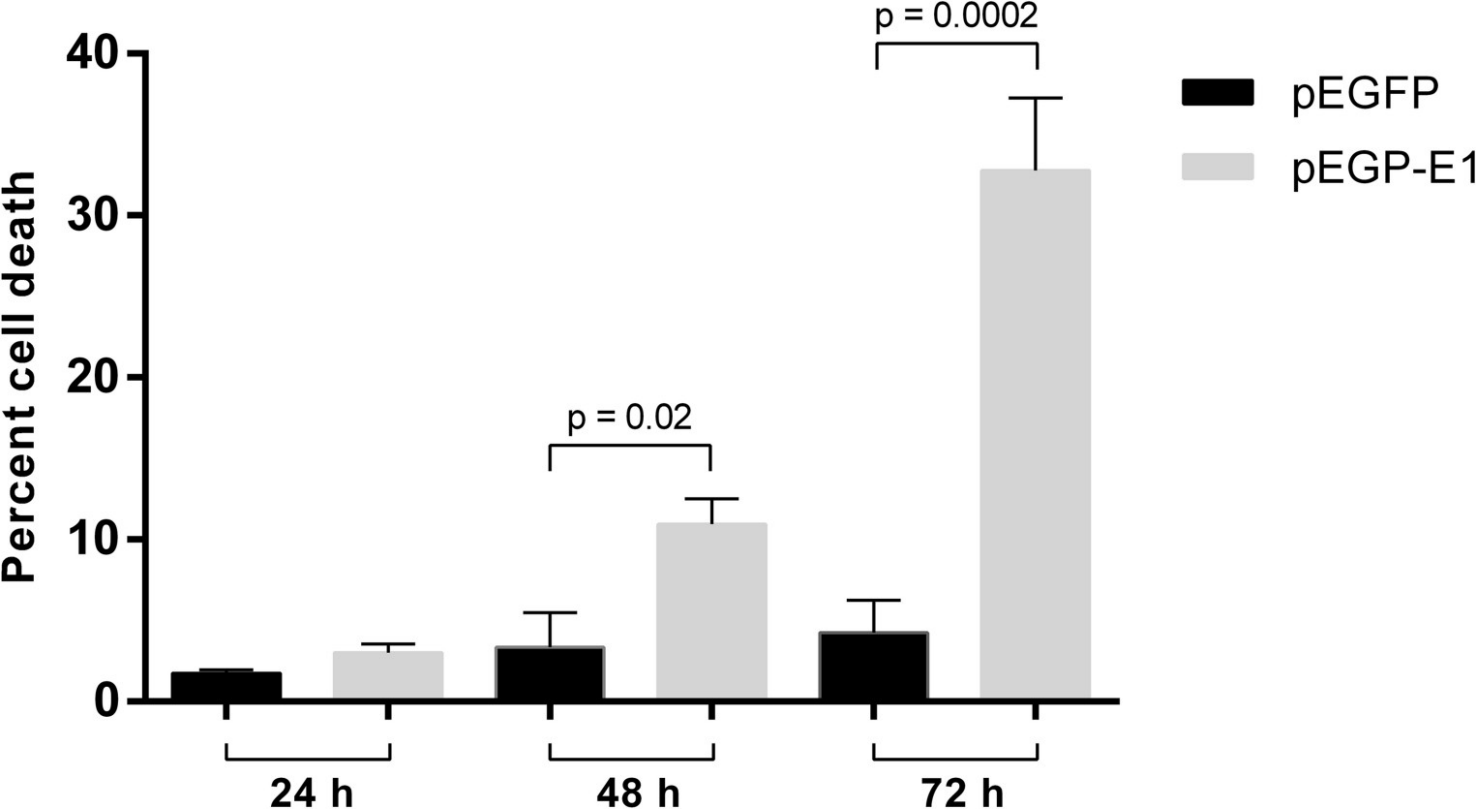
HPV16 E1 induces cell death. HEK 293 T cells were transfected with either pEGFP (black bar) or pEGFP-E1 (gray bar). Cells were stained with Zombie Yellow and percent cell death was analyzed by flow cytometry at 24-, 48-, and 72-hours post-transfection.

**Fig 7:**
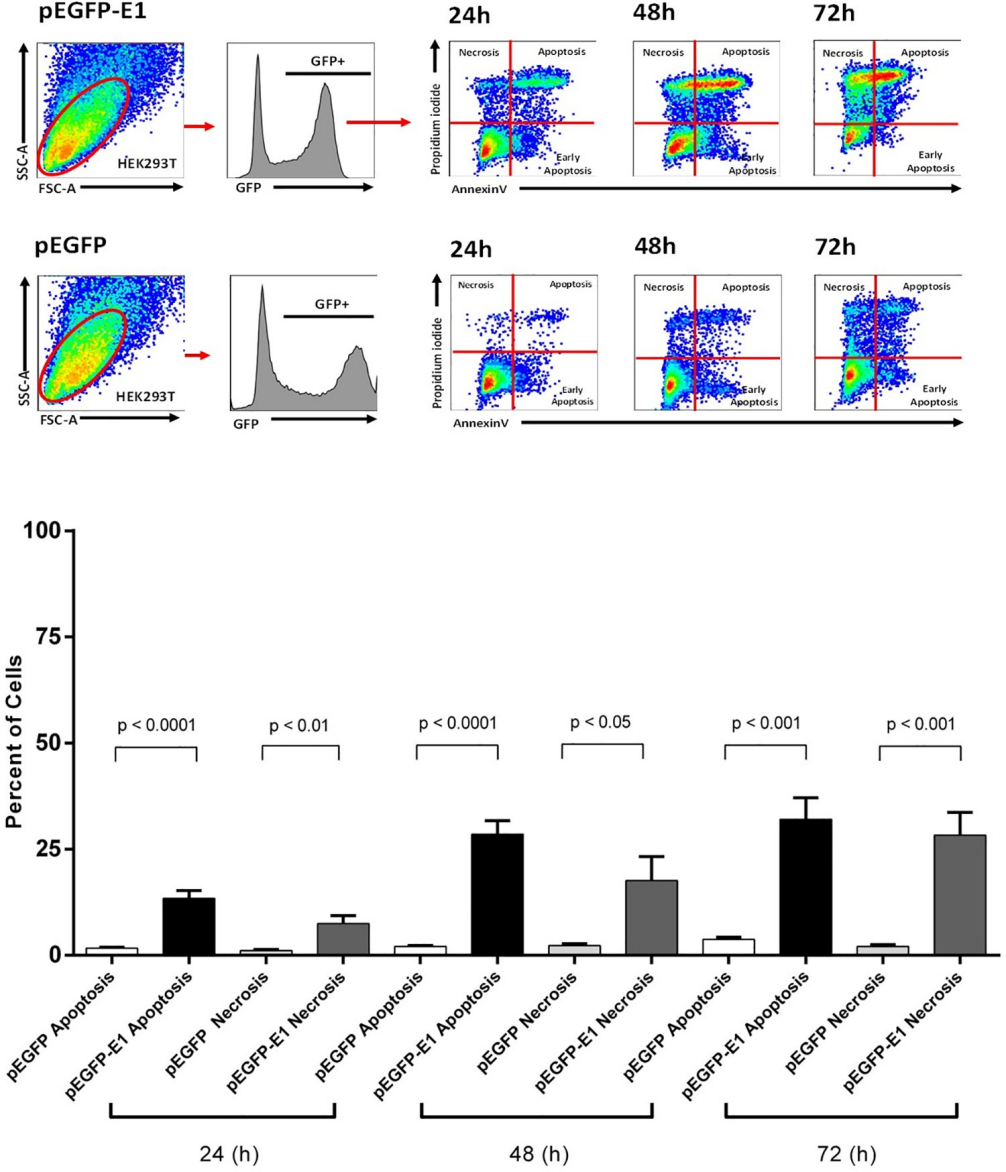
HPV16 E1 induces both apoptosis and necrosis. HEK 293 T cells were transfected with either pEGFP or pEGFP-E1. Apoptosis (annexin V staining) and necrosis (propidium iodide staining) were measured by flow cytometry. A: GFP+ HEK293T cell populations were gated. The frequencies of necrosis cells were identified as annexinV-PI+, apoptosis cells were identified as annexinV+PI+ and early apoptosis cells were identified as annexinV+PI-. B: Mean percentage of apoptosis and necrosis from 3 independent experiments were shown with statistically significance at *p*-value<0.05.

The previous experiments indicated that transfection with full-length HPV16 E1 decreased cellular proliferation, which could accompany enhanced cell death. E1 overexpression significantly induced cell death, at 48- and 72-hours post-transfection (Fig 6). In order to determine whether HPV16 E1 induced cell death through apoptosis, HEK 293T cells were transfected with either pEGFP or pEGFP-E1 and the extent of apoptosis measured by annexin V and propidium iodide staining and flow cytometry. The results indicated that HPV16 E1 significantly induced apoptosis starting at 24 (*p*-value < 0.0001) and continued throughout 48 (*p*-value < 0.0001), and 72 (*p*-value < 0.001) hours post-transfection. The difference in mean percentage of apoptotic cells between E1 transfected cells and the vector control were 11.72%, 26.44% and 28.3% at 24, 48, and 72 hours, respectively. A significant increase in mean percentage of necrotic cells was also observed, i.e., 6.33% (*p*-value = 0.01), 15.35% (*p*-value < 0.05), 26.20% (*p-*value < 0.001) at 24, 48, and 72 hours, respectively (Fig 7). This experiment demonstrated that, in addition to decrease cell proliferation, E1 also causes cell death through both the apoptosis and necrosis pathways.

Subsequently, to confirm whether cell death caused by HPV16 E1 was both apoptosis and necrosis, cells were pre-treated with QVD-OPH prior to transfection. There was a decreasing trend (*p*-value = 0.20) of apoptosis in cells treated with QVD-OPH compared to untreated cells, with a decrease in the mean percentage of apoptotic cells by 0.23% (pEGFP) and 2.42% (pEGFP-E1), thus suggesting that apoptosis plays only a part of HPV16 E1-mediated cell death.

### HPV16 E1 affects many cellular pathways

To further explore the role of HPV16 E1, microarray analysis was performed on RNA collected from E1 overexpressing cells. Cellular gene expression profile revealed that E1 impacted gene expression in numerous pathways. In total, 416 genes were differentially expressed in HPV16 E1 transfected cells. Of these 238 were upregulated (≥ 2-fold change), and 177 were downregulated (≤ 2-fold change). The genes with the highest differential expression in E1 transfected cells were small nucleolar RNA C/D box A and C, SNORD3A (16.59-fold change) and SNORD3C (16.02-fold change); and SNORD84 (7.31-fold change). The next overexpressed gene was interferon stimulated gene 20, ISG20 (8.94-fold change) which is a gene known to exhibit antiviral properties. The fifth upregulated gene was variable charged X-link, VCX (6.21-fold change). In contrast, E1 suppressed the expression of many genes including inhibin beta E subunit, INHBE (-5.70-fold change). INHBE encodes for an inhibin beta subunit which is a member of transforming growth factor-beta superfamily. In addition, DNA Damage Inducible Transcript 4, DDIT4 (-4.43-fold change), normally upregulated during DNA damage [14], TSC22 domain family member 3, TSC22D3 (-3.90-fold change), encodes a glucocorticoid induced leucine zipper protein and interacts with FoxO3 were also suppressed. The most influenced pathways induced by E1 included ribosome, metabolism, transcriptional dysregulation, cell proliferation and cell death (Table 1). Many genes involved in cell proliferation and death were significantly different between the vector control and E1 transfected cells. A total of 117 genes differentially expressed genes involved in cell growth/death were identified, 59 were upregulated while 58 were downregulated (Table 2).

**Table 1:** Pathways significantly influenced by HPV16 E1 from gene expression analysis.

Pathway	Sig.Genes	*P-*value	Pathway	Sig.Genes	*P-*value
Ribosome	14	9.33E-15	Toll-like receptor signaling pathway	4	0.00692
FoxO signaling pathway	9	4.51E-08	Alanine, aspartate and glutamate metabolism	3	0.00763
Transcriptional misregulation in cancer	9	3.75E-07	Bladder cancer	3	0.00887
MAPK signaling pathway	10	4.97E-07	Cell cycle	4	0.01049
PI3K-Akt signaling pathway	11	6.39E-07	Osteoclast differentiation	4	0.01212
Small cell lung cancer	7	9.77E-07	Amino sugar and nucleotide sugar metabolism	3	0.01362
Pathways in cancer	11	2.12E-06	Endocytosis	5	0.01427
Protein processing in endoplasmic reticulum	8	3.33E-06	Wnt signaling pathway	4	0.01442
Metabolic pathways	17	8.45E-06	Insulin signaling pathway	4	0.01442
Prostate cancer	6	0.00002	Signaling pathways regulating pluripotency of stem cells	4	0.01496
NF-kappa B signaling pathway	6	0.00002	Glutathione metabolism	3	0.01521
Viral carcinogenesis	7	0.00011	Hippo signaling pathway	4	0.01845
Glycolysis / Gluconeogenesis	5	0.00012	Acute myeloid leukemia	3	0.01863
Biosynthesis of antibiotics	7	0.00014	cGMP-PKG signaling pathway	4	0.0227
Regulation of actin cytoskeleton	7	0.00014	Pancreatic cancer	3	0.0243
Ubiquitin mediated proteolysis	6	0.00016	Central carbon metabolism in cancer	3	0.02497
Apoptosis	5	0.0003	Thyroid hormone synthesis	3	0.02842
TNF signaling pathway	5	0.00074	Biosynthesis of amino acids	3	0.02985
MicroRNAs in cancer	7	0.00076	Chemokine signaling pathway	4	0.031
cAMP signaling pathway	6	0.00084	Cardiac muscle contraction	3	0.0328
AMPK signaling pathway	5	0.00114	TGF-beta signaling pathway	3	0.03431
Prolactin signaling pathway	4	0.00243	Salmonella infection	3	0.03901
Phagosome	5	0.00252	ErbB signaling pathway	3	0.03982
RNA transport	5	0.00362	Rapl signaling pathway	4	0.04068
Estrogen signaling pathway	4	0.00592	Gap junction	3	0.04145
HIF-1 signaling pathway	4	0.00641	Ras signaling pathway	4	0.04908

**Table 2:** Genes differentially expressed in HPV16 E1 transfected cells involved in cell proliferation and cell death.

Gene	FC	Gene	FC	Gene	FC	Gene	FC
ISG20	**8.94**	GSTM3	**2.26**	BCL2	**-2.05**	ASNS	**-2.35**
SERTADI	**4.76**	CX3CR1	**2.23**	MSH5	**-2.07**	SLC11A2	**-2.35**
SGK1	**4.67**	HSPA2	**2.23**	E2F2	**-2.07**	FOXO3	**-2.36**
RGS2	**3.70**	TNFRSF10D	**2.22**	MECOM	**-2.07**	H1F0	**-2.40**
KRIDAP	**3.49**	PSMD10	**2.22**	NFKB1	**-2.09**	JARID2	**-2.45**
CD14	**3.32**	SIRT4	**2.21**	SPEN	**-2.10**	TENM3	**-2.45**
GDF15	**3.25**	NPTX1	**2.21**	PPARGC1A	**-2.10**	NCOA1	**-2.47**
BIRC5	**3.08**	EOMES	**2.20**	SATB2	**-2.10**	KLF9	**-2.48**
HBEGF	**3.06**	KIF20A	**2.19**	SASS6	**-2.10**	TCF12	**-2.50**
ALDOC	**2.97**	TP53INP2	**2.18**	ASAP1	**-2.10**	FHIT	**-2.50**
IFI27	**2.90**	KRT17	**2.17**	NSMCE2	**-2.11**	BARD1	**-2.50**
HOXDI	**2.84**	KIAA1324	**2.17**	MYC	**-2.16**	KDM6A	**-2.51**
APOBEC3H	**2.78**	EGRI	**2.16**	HEY1	**-2.16**	PSD3	**-2.53**
TEX19	**2.66**	NMB	**2.13**	WDR7	**-2.19**	NGFRAP1	**-2.54**
FGFR3	**2.63**	THBS4	**2.12**	MKL1	**-2.19**	NFIB	**-2.55**
BAX	**2.63**	SLC9A3R1	**2.12**	ARID4B	**-2.21**	MEIS2	**-2.56**
PLK5	**2.62**	ZNF268	**2.12**	CTH	**-2.22**	FANCG	**-2.60**
LGALS1	**2.62**	LTA	**2.11**	FBXW7	**-2.23**	KLHL9	**-2.63**
ITGB2	**2.53**	FSCN1	**2.10**	FAF1	**-2.25**	RARB	**-2.65**
GADD45B	**2.49**	RPS15	**2.09**	FAM188A	**-2.25**	MAP2K5	**-2.67**
GAL	**2.47**	PGK1	**2.09**	HILPDA	**-2.25**	PBX3	**-2.73**
PLK2	**2.45**	SOX8	**2.09**	NFKBIA	**-2.25**	ARID5B	**-2.83**
CDK5R1	**2.43**	TAF7L	**2.08**	BCL11A	**-2.28**	RAPGEF6	**-2.93**
SLC9A1	**2.42**	PKNOX1	**2.08**	ATG7	**-2.29**	TSC22D3	**-3.25**
SOD2	**2.41**	HES5	**2.07**	RAD51D	**-2.31**	EXT1	**-3.33**
EVA1A	**2.41**	METRNL	**2.03**	PBX1	**-2.31**	DDIT4	**-4.43**
TCP1	**2.30**	CDKN2D	**2.02**	BTRC	**-2.33**	INHBE	**-5.70**
GABPA	**2.29**	BCL7C	**2.01**	NUPR1	**-2.34**		
CACYBP	**2.29**	PTH1R	**2.01**	SSH2	**-2.35**		

FC: Fold change.

**Fig 8:**
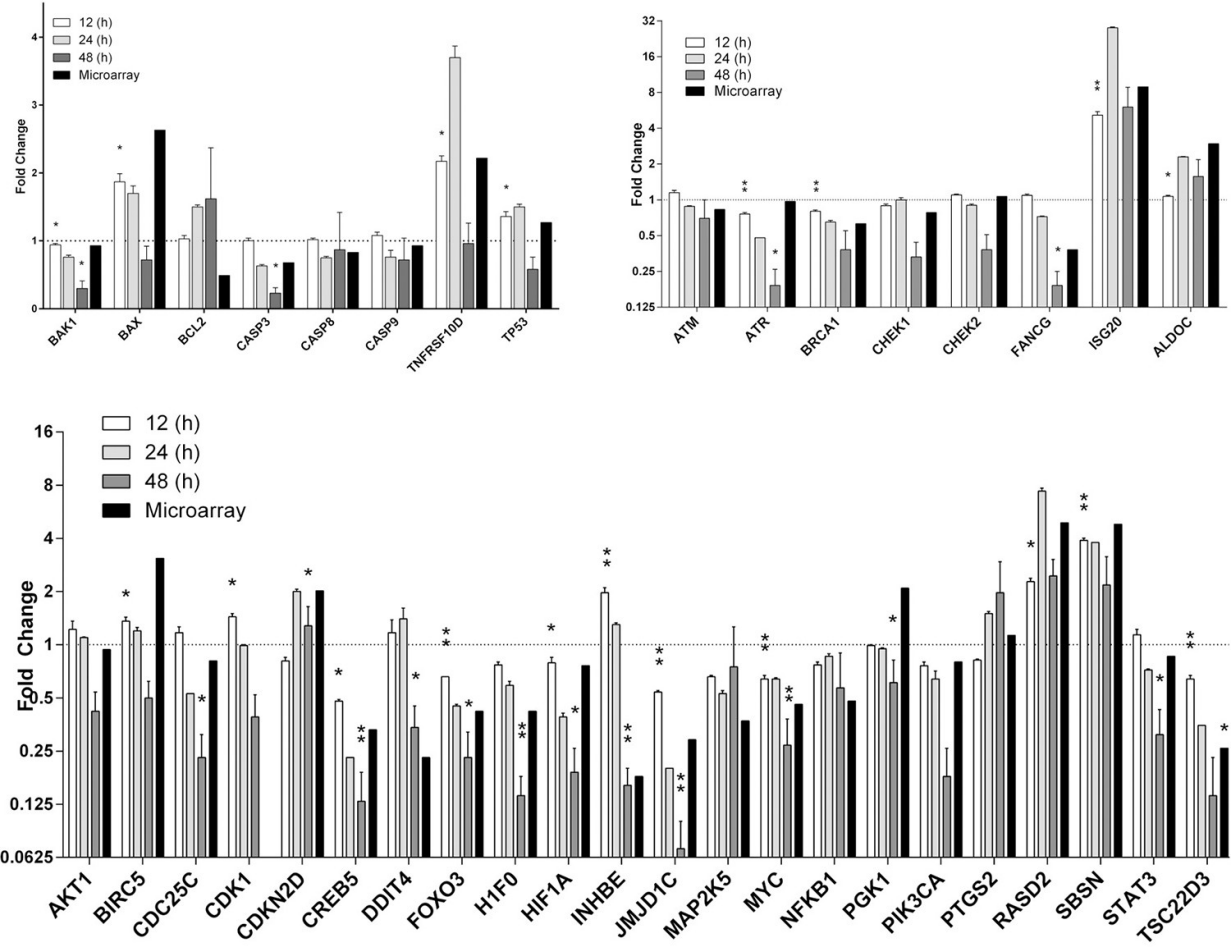
HPV16 E1 impacts host gene expression. Quantitative real-time RT PCR was performed on pEGFP and pEGFP-E1 transfected cells. Fold change between pEGFP and pEGFP-E1 is displayed. A: Apoptosis regulating genes. B: DNA Damage, immune response and metabolism regulating genes C: Cell proliferation regulating genes (*p*-value < 0.05 denoted by * and < 0.01 by **).

Because E1 influences many pathways involved in cell death and proliferation, a panel of hallmark genes in cell proliferation, apoptosis, and DNA damage pathways, along with genes that were highly up/downregulated from the microarray analysis were used for validation by real-time RT PCR. A total of 38 genes were included in the panel (Fig 3). In total, the real-time RT PCR identified 24 differentially expressed genes (3 upregulated and 21 downregulated) after 48 hours of transfection. The genes most affected by E1 transfection were genes involved in cell proliferation and DNA damage. Only 2 out of 8 genes, BAK1 (0.30) and CASP3 (0.23), in the apoptosis pathway showed any differential expression. Whereas 16 out of 22 in the cell proliferation and 5 out of 6 genes in the DNA damage pathways were found. Once it was confirmed that E1 had a significant effect on gene expression at 48 post-transfection the next experiment sought to determine if the effect on gene expression was time dependent. Therefore, real-time RT PCR was performed on RNA collected from cells transfected with either vector control or HPV16 E1 at 12 and 24 hours post transfection. The results revealed that changes in expression occurred in a time-dependent manner. At 12 hours post-transfection only 5 genes out of the 38 in the panel were differentially expressed, while 12 genes were up/downregulated after 24 hours of transfection. The results from the real-time RT PCR concluded that E1 has an effect on gene expression in a time-dependent manner, however it was noted that most genes were down regulated (Fig 8).

## Discussion

In this study, we demonstrate that HPV16 E1 expression negatively influences cell proliferation and viability by approximately 2-fold within 24 hours post-transfection (Fig 2). A similar effect has been demonstrated in a previous study using HPV31 E1 [15], but in that case, the decreased cell proliferation occurred only 3 weeks post transfection. We further attempted to characterize the domain(s) of E1 that impacted cell proliferation and confirmed that only full-length HPV16 E1 was able to significantly decrease cell proliferation (Fig 5). This observation is in agreement with another independent study [9].

The decrease in cell proliferation or cell growth can be induced by cell cycle arrest and/or cell death. Death of E1 expressing cells increased relative to time, post-transfection (Fig 6). Our results revealed that E1 expressing cells induced both apoptotic and necrotic cell death, with a slightly higher incidence of apoptotic cell death (Fig 7), however apoptotic cell was only reduced slightly when treated with QVD-OPH. This could be attributed to enhanced binding of annexin V to the necrotic cells, owing to disruption of cell membranes. One proposed explanation for the induction of cell death in transfected cells could be that the nuclear accumulation of E1 was deleterious to cell growth [9]. Moreover, a previous study by Moody et al. [16] found that HPV31 activates the apoptosis pathway, specifically, caspase 3, 7 and 9 upon differentiation to promote viral genome amplification. This activating process was induced by HPV31 E1 containing a caspase 3/7 cleavage site which is conserved among HPV including HPV16. This caspase cleaved E1 significantly increased E1 mediated transient replication of origin containing plasmids [16]. A separate study demonstrated that HPV18 E1 induced double stranded DNA breaks, and studies have shown that HPV recruits proteins involved in the ATM DNA damage pathway to nuclear foci, and that activation of ATM is required for viral genome amplification [17–19]. Another explanation that may have contributed to the amount of cell death observed in pEGFP-E1 transfected cells is the overexpression of E1 due to cytomegalovirus promoter (CMV), which is a strong promoter [20]. Effect of protein overexpression may cause lower tolerance for transported proteins in the transfected cells; proteins that need transport, like E1 may overload the cell, possibly leading to premature cell death [20]. HPV31 E1 overexpression has also been reported to cause S-phase cell cycle arrest, leading to reduced cell growth [9]. In combination, these studies illustrate that HPV E1 proteins induce DNA damage and restrict cell growth to recruit DNA repair proteins and facilitate viral replication.

Since several studies have characterized the ability of E1 to interact with various host cell proteins, microarray analysis was performed on HPV16 E1-expressing cells, in order to establish the role of E1 in regulation of gene expression in the infected host cells. Microarray results revealed that E1 significantly altered gene expression in many pathways, such as ribosome biogenesis, MAPK, PI3K-Akt, FoxO, NF-kappa B, and apoptosis signaling pathways (Table 1). A total of 14 genes were increased in the ribosome pathway. The increased expression of ribosomal proteins in E1 overexpressed cells signified an increase in protein synthesis. In addition to protein synthesis, numerous ribosomal proteins also have extra-ribosomal functions [21]. For instance, RPL36A, which was overexpressed in the microarray analysis, is upregulated in hepatocellular carcinoma and has been shown to increase cell proliferation [22]. In addition to the upregulation of ribosomal proteins, a family of noncoding RNAs involved in ribosome biogenesis, termed snoRNAs, were highly upregulated following expression of E1. Pathways involved in cell proliferation and carcinogenesis were also influenced by E1. For example, many genes in the MAPK, PI3K-Akt, FoxO, NF-kappa B, and apoptosis signaling pathways were dysregulated.

The ability of E1 to alter host gene expression in a time-dependent manner was further confirmed by RT real-time PCR. Of these, genes such as ISG20, TNFRSF10D, SBSN and RASD2 peaked at 24 hours post-transfection (Fig 8). ISG20 belongs to a group of interferon-stimulated genes, was the most intensively upregulated gene by E1 (28-fold at 24 hours and 6-fold at 48 hours). This was expected as, increased production of interferons (IFNs) in response to viral infections stimulate genes that combat infection. TNFRSF10D also known as Decoy Receptor 2 (DcR2) is a member of the tumor necrosis factor receptor superfamily, which cannot induce apoptosis because it lacks the death domain. Cervical cancer cells with decreased decoy receptor expression demonstrate high activation of death ligand-mediated apoptosis [23]. The data from the RT real-time PCR and microarray analysis indicates that E1 elevates the expression TNFRSF10D mRNA to promote cell survival. Another gene upregulated by E1 is SBSN which codes for a novel oncoprotein, Suprabasin. Suprabasin has been implicated as an oncoprotein in highly invasive glioblastoma and esophageal cancer. Mice inoculated with cells overexpressing suprabasin demonstrated enhanced tumor growth compared to the vector control, by enhancing Wnt/β-catenin signaling [24]. Similarly, upregulated RASD2 activates Akt and aids in cellular proliferation [25]. Interestingly, mRNA expression of TNFRSF10D, SBSN and RASD2 was increased at 12 and 24 hours and declined at 48 hours (Fig 8) suggesting the level of E1 protein may influence host mRNA expression.

E1 also suppressed several genes, such as CREB5, HIF1A, NFKB1 and PIK3CA. Interestingly, CREB5, HIF1A, NFKB1 belong to families of transcriptional factors. Reduction of proteins encoded by NFKB1 and PIK3CA have been shown to aid in tumor survival and growth [26]. HPV16 E1-mediated suppression of Nuclear factor-κB Subunit 1 (NFKB1) has also been shown to result in tumor growth [27]. The most downregulated gene by E1 was INHBE which codes for a preprotein which is processed to form the beta E subunit of either inhibin or activin. Both proteins are known to regulate a wide range of cellular processes including apoptosis, growth and immune response [28].

A series of genes involved in the DNA damage pathway, namely JMJD1C, ATR, BRCA1 and CHEK1 also appear to be suppressed by HPV16 E1. This is not in agreement with recent studies that report E1-mediated increase in the expression levels of DNA damage response proteins, such as ATM, ATR, CHK1 and CHK2 [29]. The tumor suppressor protein, BRCA1 [30] and a histone demethylase called Jumonji Domain Containing 1C (JMJD1C), commonly reduced or lost in breast cancer [31] are also suppressed in the E1-expressing cells. Genes such as CDC25C, DDIT4, and INHBE, all of which are involved in the DNA damage pathway, are also suppressed following E1 overexpression. INHBE was the most downregulated gene by E1 and has been shown to regulate a wide range of cellular processes including apoptosis, growth and immune response [28].

Glucocorticoid-induced leucine zipper (GILZ) protein encoded by the TSC22D3 gene is associated with the mTOR2C/AKT signaling cascade [32]. GILZ can also activate FOXO3 transcription of the pro-apoptotic protein, Bim [32]. Our study illustrated that E1 suppressed the expression of GILZ by more than 7-fold, and thus prevented the activation of FOXO3, which in turn is linked to cell survival and growth [33]. Numerous studies have been conducted on the relationship of FOXO proteins and tumorigenesis [34]. For example, a study examined colorectal cancer patients and found that FOXO3 expression was reduced in tumor tissue samples. Low FOXO3 expression was also correlated to decreased patient survival [35].

Altogether, E1 altered expression of certain genes to support cell survival and growth between 12–24 hours post-transfection. As it is well known that E1 is expressed very early after HPV infection, it is possible that genes induced by E1 are essential to support the viral amplification. During HPV infection, the episomal form of HPV 16 genome represents active viral replication while there is no active replication once the viral genome is integrated into the host genome [36, 37]. Although the role of E1 in carcinogenesis is not known, E1 expression levels obtained from episomal HPV16 positive patients showed higher levels than from cell lines with integrated form. HPV16 E1 expression increased with cervical cancer progression at 0.18 (Normal), 0.42 (CIN 1), 0.65 (CIN 2/3) and 0.79 (SCC) whereas E1 expression in HPV-16 positive cervical cell lines such as CaSki (0.13) and SiHa (0.06) cells were observed [12]. Moreover, the expression of E1 does not depend on the HPV DNA copy number (CaSki approximately 600 copies, SiHa 1–2 copies per cell). From our previous data, a low level of E1 is essential for maintaining the cancer stage.

## Conclusion

Collectively, gene expression analysis following E1 transfection signified its importance in host gene expression and pathway regulation. E1 not only increased the expression of genes involved in cell survival, but it also induced cell death. It is generally believed that carcinogenesis occurs as a result of evasion of apoptosis [10]. However, a recent study suggested that apoptosis drives carcinogenesis by sending survival signals to surrounding cells [38]. For example, breast-, colorectal-, non-small cell lung-cancer patients with high expression of the anti-apoptotic protein BCL-2 are associated with favorable prognoses [39, 40]. This suggests that HPV16 E1 may also induce carcinogenesis in a similar manner, owing to its high expression levels in cervical cancer patients.

Our study used HEK293T cells instead of keratinocytes or cervical cancer cells to ensure the efficiency of transfection. We believe that the effect of E1 protein in HEK293T cells would not be cell-type specific since several studies on HPV16 oncoprotein also used HEK293T cells [41, 42]. Although our data contradicts some previous data [43], we would like to note that this may be due to the deleterious expression of E1 after 48 hours post transfection. This deleterious expression most likely leads to a higher-than-normal amount of cell death, which contradicts previous reports and may mask the true effects of E1. This observation warrants further studies.

## Supporting information

S1 File (XLSX)

S2 File (XLSX)

S3 File (XLSX)

S4 File (XLSX)

S5 File (XLSX)

S6 File (XLSX)

S7 File (XLSX)

S8 File (XLSX)

S9 File (XLSX)

S10 File (XLSX)

S11 File (XLSX)

S12 File (XLSX)
